# Significance of serum galactose deficient IgA1 as a potential biomarker for IgA nephropathy: A case control study

**DOI:** 10.1371/journal.pone.0214256

**Published:** 2019-03-27

**Authors:** Soumita Bagchi, Raghavendra Lingaiah, Kalaivani Mani, Adarsh Barwad, Geetika Singh, Veena Balooni, Dipankar Bhowmik, Sanjay Kumar Agarwal

**Affiliations:** 1 Department of Nephrology, All India Institute of Medical Sciences, New Delhi, India; 2 Department of Laboratory medicine, All India Institute of Medical Sciences, New Delhi, India; 3 Department of Biostatistics, All India Institute of Medical Sciences, New Delhi, India; 4 Department of Pathology, All India Institute of Medical Sciences, New Delhi, India; International University of Health and Welfare, School of Medicine, JAPAN

## Abstract

**Background:**

IgA nephropathy(IgAN) is a common glomerular disease with a higher risk of progression to end stage renal disease (ESRD) in certain ethnic populations. Since galactose deficient IgA1(Gd-IgA1) is a critical molecule in its pathogenesis, it has generated interest as a biomarker for this disease.

**Methods:**

We measured serum Gd-IgA1 levels using a non- lectin based enzyme linked immunoassay(ELISA) in 136 immunosuppression naïve patients with primary IgAN and 110 controls(60-non IgA glomerular diseases, 50-healthy volunteers).

**Results:**

Median serum Gd-IgA1 levels were significantly higher in IgAN patients [13135.6(2723.3,59603.8)ng/ml] compared to those with non IgA glomerular disease [4954.8(892.9,18256.2) ng/ml] and healthy controls [6299.5(1993.2,19256) ng/ml] and this was observed even after log transformation and adjustment for age and gender(p<0.0001). Considering a cut-off value of serum Gd-IGA1≥7982.1ng/ml, the sensitivity for diagnosing IgAN compared to healthy controls was 74.3% and specificity was 72.0% with a positive predictive value of 87.8% and negative predictive value of 50.7%. The serum Gd-IgA1 level did not co-relate with baseline estimated glomerular filtration rate, urine protein creatinine ratio and the M, E, S, T and C scores on renal biopsy. The renal survival (absence of >30% decrease in eGFR, ESRD or death) was lower in patients with higher serum Gd-IgA1 levels(≥7982ng/ml) than those who had lower levels but it was not statistically significant(p = 0.486).

**Conclusion:**

Serum Gd-IgA1 level is higher in IgAN patients compared to non-IgA glomerular diseases and healthy controls and has a good positive predictive value for diagnosis. However, it does not correlate with clinical and histological characteristics of disease severity and does not predict disease progression.

## Introduction

IgA nephropathy (IgAN) is the most commonly reported primary glomerular disease in adults. It has a wide clinical spectrum ranging from isolated microscopic/macroscopic hematuria, subnephrotic proteinuriato a heavy proteinuric illness and/or declining renal function. Upto 30–40% of patients with IgAN progress to End Stage Kidney Disease (ESKD) by 20 years [1,2]. It is reported to have a more aggressive clinical course with poor renal survival in the Indian population [3–6].Renal biopsy with immunofluorescence is essential for the diagnosis of IgA nephropathy. Over the years, research has focused on establishing a biomarker for this disease [7–11] to help us in diagnosis and also monitoring the clinical course, particularly in patients with mild disease.

Galactose deficient IgA1 (Gd-IgA1) is a critical molecule in the pathogenesis of IgAN [7–10]. The O-linked glycans in the hinge region of IgA1 are generally composed of N-acetyl galactosamine (GalNAc) and galactose with sialic acid may be attached to either or both sugars. Gd-IgA1 acts as an antigen, combines with autoantibodies to form immune complexes which get deposited in the mesangium and stimulate downstream action. Gd-IgA1 containing immune deposits and mesangial cell proliferation are characteristic features of IgAN[11].Significantly higher levels of circulating IgA1 with galactose-deficient, O-linked, hinge-region glycans have been reported in IgA nephropathy patients compared to non-IgA renal disease and healthy controls in Caucasians, African Americans, Japanese and Chinese populations [12–17]. This has led to an interest in this molecule as a potential biomarker for IgAN. A snail helix aspersa agglutinin (HAA) lectin based ELISA assay has been used to measure serumGD-IgA1 in patients in these studies. Its widespread use in clinical practice has been hindered by certain limitations. The bioactivity and stability of this assay depends on the product lot of HAA lectin and it is also difficult to procure HAA lectin appropriate for this assay. Also, it is a complex procedure thereby restricting its use to specialized laboratories.

A research group in Japan has developed a lectin independent ELISA assay using a unique anti-Gd-IgA1 monoclonal antibody KM55 to overcome the limitations of the lectin based assay [18]. The KM55 antibody is procured steadily from hybridoma cells. The antibody is specific to a glycoform of Gd-IgA1 which may be overemphasized by this assay. This technique has been validated against the lectin based assay and found to be a robust assay for detecting serum GD-IgA1[18].However studies using this assay in IgAN patients are lacking.

There is no data about Gd-IgA1 in the Indian patients with IgAN. We conducted this study to assess the efficacy of serum Gd-IgA1 as a biomarker for diagnosis of IgAN and to determine its correlation with the severity of the disease.

## Method

In a case control study we compared serum Gd-IgA1 levels in patients with primary IgAN and controls (non IgA glomerular disease and healthy volunteers).The study was approved by the Institute Ethics Committee, All India Institute of Medical Sciences, New Delhi (IEC-125/07.04.2017,RP-47/2017). Informed written consent was taken from all the patients and controls. If the participant was less than<18 years age, then the consent was taken from the parent/guardian.

136 patients with biopsy proven primary IgAN seen in our clinic from March 2013- August 2018 were included in the study. 60 patients with other biopsy proven non-IgA glomerular diseases diagnosed during the same period and 50 healthy volunteers were taken as controls. The number of participants in each group was based on the feasibility determined by the scope of the funding available.Gd-IgA1 level was assessed in the sera of patients as well as both controls groups. Baseline sera of patients with IgAN(cases) and non IgA glomerular disease (controls) was obtained from a biorepository (IEC/NP-259/2013 & RP-24/05.08.2013) where we store serum samples of patients collected at the time of renal biopsy with their consent since 2013. Sera of the healthy volunteers were collected prospectively in 2018. Samples were stored at -800C until analysis with minimal freezing and thawing.

We excluded patients who(1) did not give informed consent, (2) had history of immunosuppression use before sample collection (3)had secondary causes of IgAN like chronic liver disease (4) Henoch-Schonlein purpura (5) a second coexisting disease on kidney biopsy like diabetic nephropathy (6) inadequate/missing clinical records and (7) inadequate kidney biopsy.

All the study patients were seen in the renal clinic and clinical and histopathologic data were collected ambispectively. Details of laboratory investigations done for these patients as part of their regular evaluation for glomerular disease were recorded. This included serum creatinine, serum albumin and serum cholesterol and urinary protein creatinine ratio(UPCR). Estimated glomerular filtration rate(eGFR) was calculated using the 4 variable Modification of Diet in Renal Disease(MDRD) formula for adults and the revised Schwartz formula for those who were <18 years of age. The primary end point was sustained >30% irreversible decline in eGFR from the time of diagnosis, end stage kidney disease(ESKD) or death whichever occurred first.

The MEST-C score of IgA nephropathy was recorded in each patient as per the Oxford classification of IgAN [19]. The study was funded by a grant from All India Institute of Medical Sciences, New Delhi and approved by the Institute Ethics Committee.

### Measurement of serum galactose deficient IgA1(Gd-IgA1) level

Gd-IgA1 level in serum was measured by solid phase sandwich enzyme immunoassay kit (IBL international GmBH, Germany) following the manufacturer’s instructions. The samples were initially diluted 200 fold with the EIA buffer to obtain optical density within the measurement range of the kit (1.56 ~ 100 ng/ml). Some samples with high optical density beyond the linearity, were reanalyzed with serial higher dilutions for accurate results. All the samples were tested in duplicates and the mean values were used for analysis.

The serum Gd-IgA1 values of IgAN patients were compared to that of the controls to assess the efficacy of this molecule as a diagnostic biomarker for IgAN.

Total serum IgA levels were not measured in this study, so we did not normalise the serum Gd-IgA1 level to the total IgA level.

## Statistical analysis

Stata 12.0 (College Station, Texas, USA) was used for analysis. Data were summarized as frequency(%), mean ± SD or median(range). The Chi square test was used to compare the categorical variables between the groups. The Wilcoxon rank sum test/t test were used to compare continuous variables between two groups depending on the normality of its distribution in the population. The one way Anova and Kruskal Wallis analysis of variance were used to compare the continuous variables between the 3 groups(IgAN, non IgAN glomerular disease and healthy controls). The receiver operating characteristic curve for serum Gd-IgA1 level was constructed and based on that the sensitivity and specificity of this assay as a diagnostic test for IgA nephropathy was calculated. Normality of the serum Gd-IgA1 was tested, and log transformation was done as it was not normally distributed. Comparison of serum Gd-IgA levels was done between the three groups using linear regression analysis adjusting age and gender. The Spearman correlation coefficient was used to assess the relationship between serum Gd- IgA1 levels and estimated GFR and urine protein creatinine ratio at the time of biopsy. Renal survival was calculated using the Kaplan-Meier analysis and risk for progression using Cox regression analysis.

## Results

### Clinical characteristics of patients and controls

The mean age of the patients with IgAN was 31.9±9.5 and 70.6% were males. Their baseline eGFR was 58.2±44.2 ml/min/1.73m2, urine protein creatinine ratio was 3.7±0.9 g/g and serum albumin was 3.7±0.9 g/dl. The mean age of controls with non-IgA glomerular diseases was 36.0±14.2 years and the healthy controls was 41.8±8.7 years with the proportion of males being 66.7% and 44% respectively. The healthy controls were older(p<0.001) with a female predominance(p = 0.003) compared to both the IgAN group and the non-IgA glomerular disease control group.

### Histological characteristics of patients and controls

On the basis of Oxford classification of IgAN, 86% patients had mesangial hypercellularity (M1), 7.4% had endocapillary hypercellularity (E1), 68.4% had segmental glomerulosclerosis (S1), 41.9% had tubular atrophy/interstitial fibrosis (T1/T2, T1-27.9%, T2-14.0%) and 15.4% had crescents(C1/2, C1-11.0%, C2-4.4%)

The renal biopsy diagnosis in the 60 controls with non IgA glomerular diseases were: minimal change disease(MCD)/focal segmental glomerulosclerosis (FSGS):25, membranous nephropathy (MN):25 and lupus nephritis:10.

### Serum Gd-IgA1 levels of patients and controls

**Fig 1:**
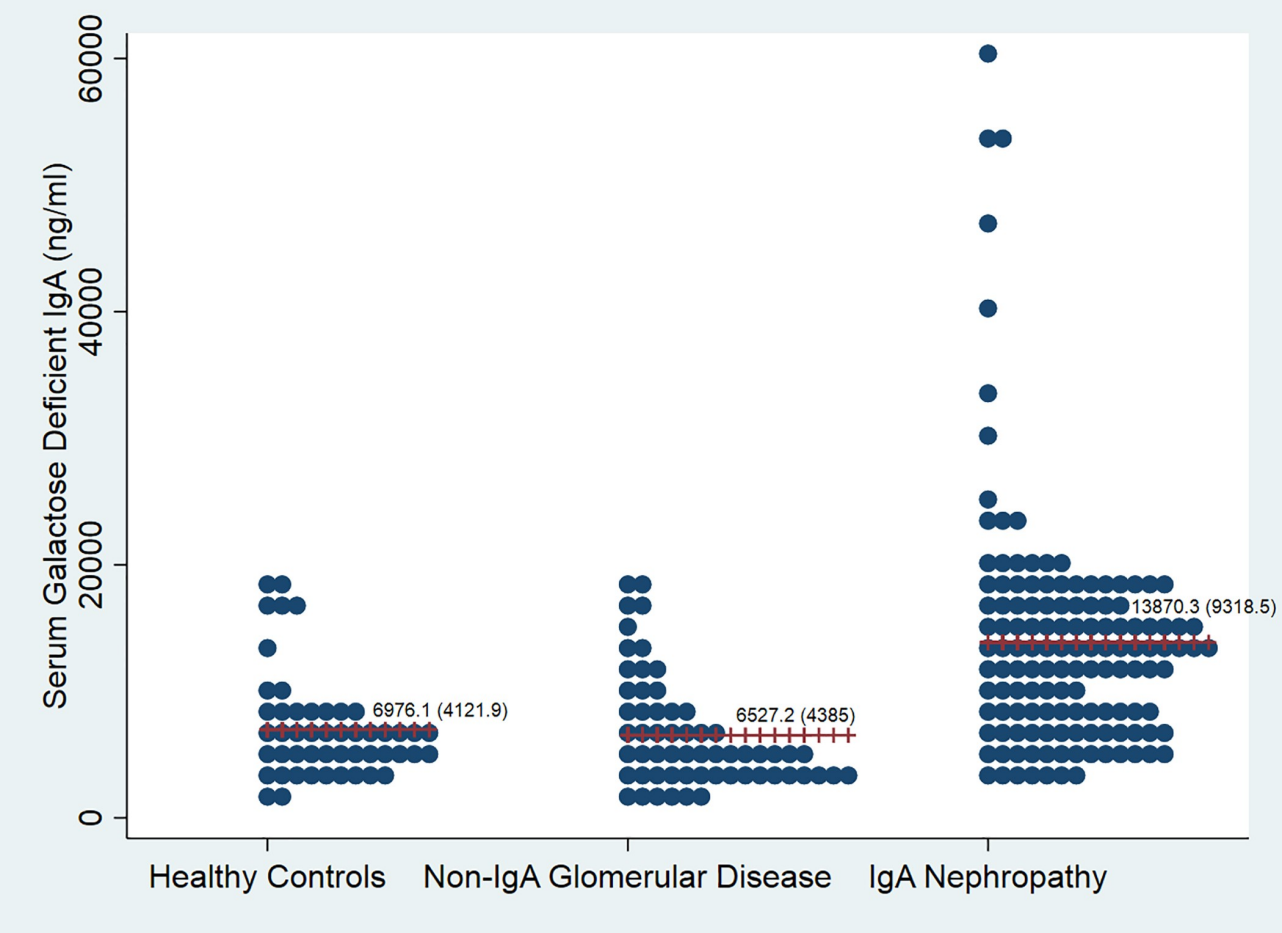
Dotplot showing individual serum galactose deficient IgA1 values in all three groups.

**Fig 2:**
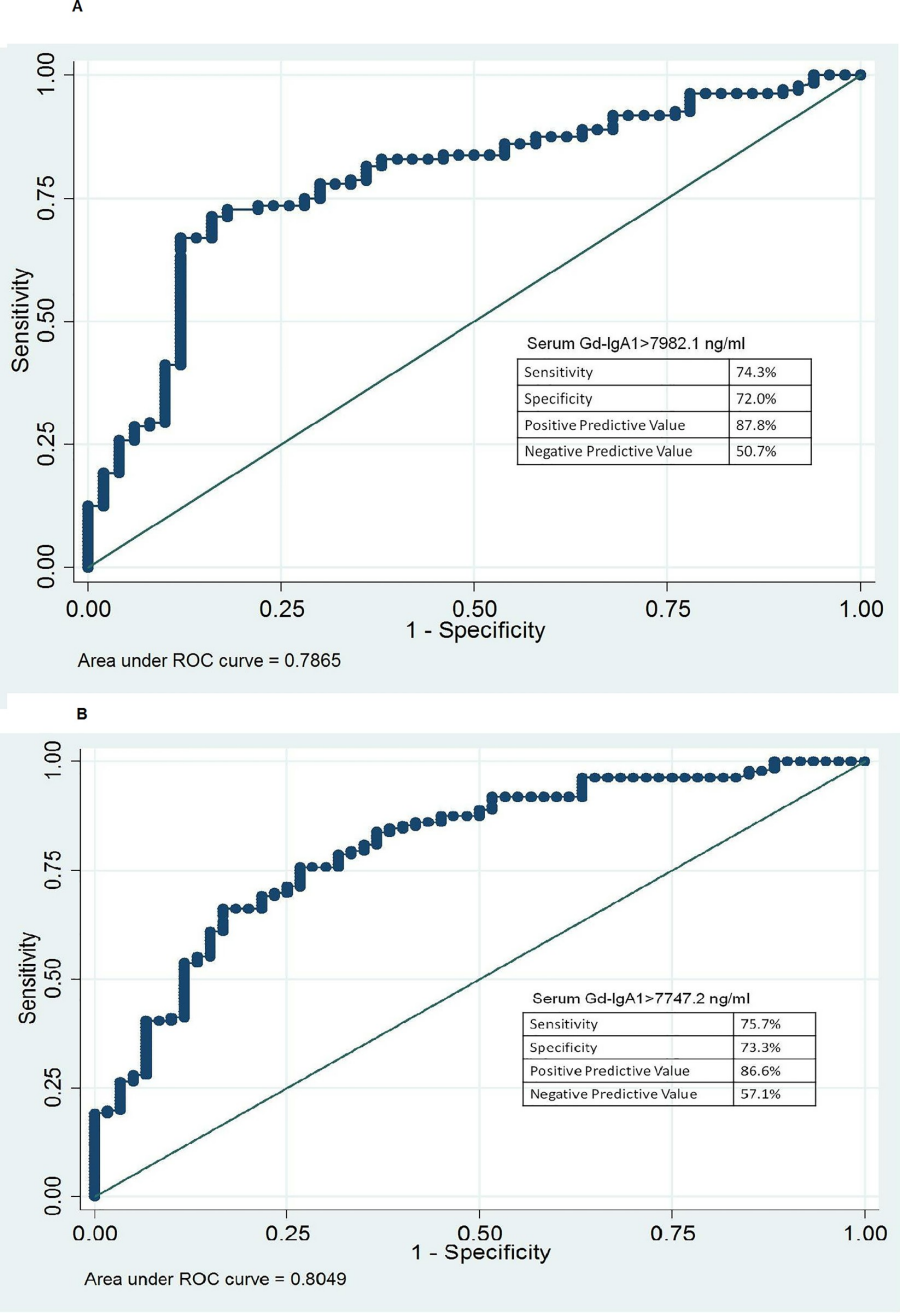
A ROC curve of serum galactose deficient IgA1 level against IgA nephropathy patients and healthy controls 2B ROC cure of serum galactose deficienty IgA1 level against IgA nephropathy patients and non-IgA glomerular disease controls.

Mean and median values of serum GD-IgA1 in all the groups are shown in Table 1 and Fig 1. Serum Gd-IgA1 levels were significantly higher in patients with IgAN compared to both the control groups(p<0.0001). Based on the receiver operating characteristic(ROC)curve for serum Gd-IgA1 levels of patients and healthy controls, considering optimum sensitivity and specificity,(Fig 2A),a cut-off value of serum Gd-IgA1≥7982.1ng/ml was *selected*. *At* this level, the sensitivity of the assay for diagnosing IgAN was 74.3% and specificity 72.0% with a positive predictive value of 87.8% and negative predictive value of 50.7%.

**Table 1:** Serum galactose deficient IgA1 levels in patients and controls.

	IgA nephropathy (n = 130)	Non-IgA glomerular disease (n = 60)	Healthy controls (n = 50)	P
Age(years), (Mean ± SD)	31.9±9.5	36.0±14.2	41.8±8.7	<0.0001
Gender(Males)(%)	96(70.6)	40(66.7)	22(44.0)	0.003
Serum Galactose deficient IgA1(ng/ml) (Mean±SD) (Median(min, max)	13870.25±9318.5, 13135.6(2723.3,59603.8)	6527.2±4385.0, 4954.8(892.9,18256.2)	6976.1±4121.9, 6299.5(1993.2,19256.0)	<0.0001

**Fig 3:**
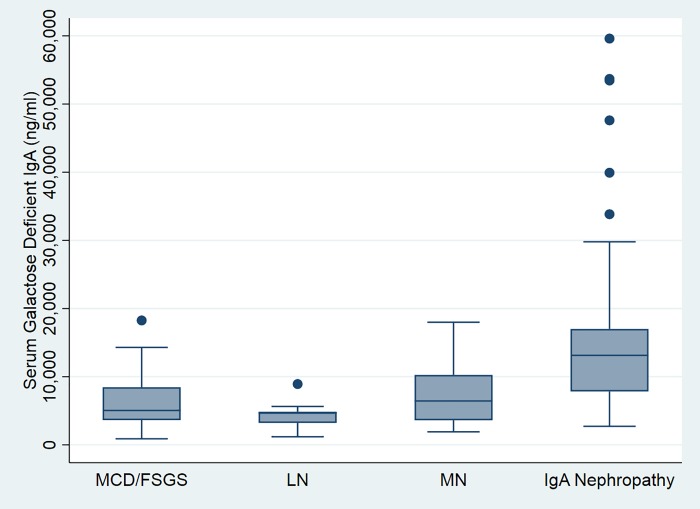
Comparison of serum galactose deficient IgA1 levels between non-IgA glomerular disease subtypes and Iga nephropathy.

The area under the curve (AUC) was 0.7865(95% confidence interval-0.71294, 0.86015) with a standard error of 0.0376. The ROC curve for patients with IgAN and controls with non-IgA glomerular diseases (Fig 2B) had an AUC of 0.8049(95% confidence interval-0.7386, 0.8712) with a standard error of 0.0338. Based on this, a cut-off of Serum Gd-IgA1>7747.2ng/ml had a sensitivity of 75.7% with a specificity of 73.3% with a positive predictive value of 86.6% and 57.1%.There was no significant difference (p = 0.959) between the mean serum Gd-IgA1 levels of the eight IgAN patients who were<18 years old (14035.04±5039.721ng/ml) and the remaining 128 patients who were ≥18 years old (13859.9 ±9534.3 ng/ml). As shown in Fig 3, there was no significant difference (p-0.2497) in the serum Gd-IgA1 levels of patients with MCD/FSGS(6257.1±4248.6 ng/ml), lupus nephritis (4268.6±2310.2ng/ml) and membranous nephropathy(7568.8±4827.5 ng/ml).

**Fig 4:**
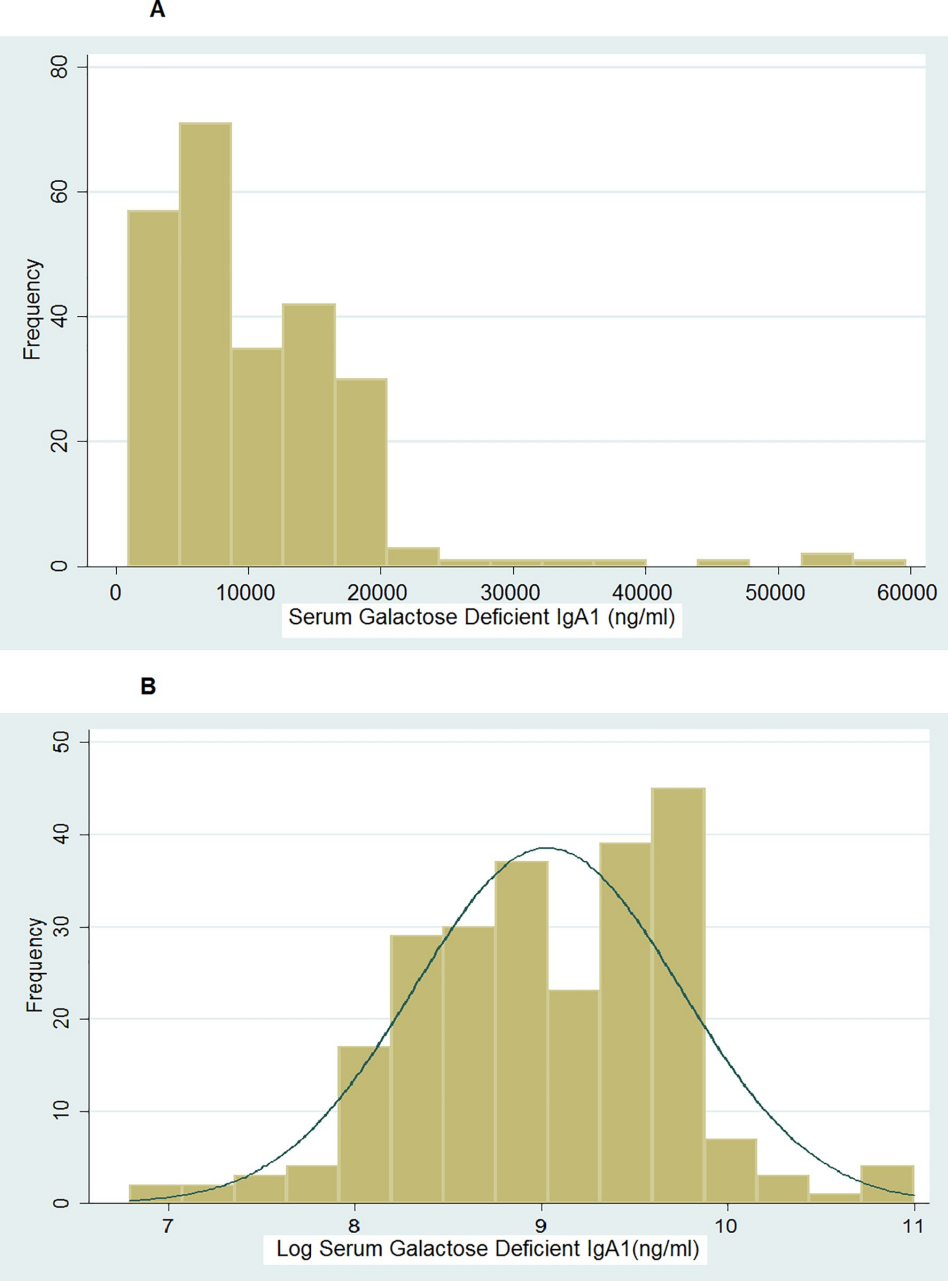
Histogram showing distribution of values of serum galactose deficient IgA1(ng/ml) before(4A) and after(4B) logarithmic transformation.

The assay levels showed significant askew in the patients as well as controls (Fig 4A). To normalize the distribution, we did the log transformation of the dataset (Fig 4B). The level of the biomarker was significantly higher in the patients compared to the controls even after log transformation and adjustment for differences in age and gender when compared by linear regression analysis (Table 2).

**Table 2:** Comparison of serum Gd-IgA1 levels among the cases and controls by linear regression analysis.

	IgA nephropathy Geometric mean(95% CI)	Non IgA disease Geometric mean(95% CI)	Healthy controls Geometric Mean(95% CI)	P
Unadjusted values	11588.1 (10454.3,12844.9)	5234.8 (4374.7 6264.0)	6053.4(5212.1 7030.5)	
Values adjusted for age and gender	11636.8 (10456.8,12956.6)	5219.0 (4457.8, 6101.4)	6007 (5001.0, 7215.4)	<0.0001

### Serum Gd-IgA1 levels and disease severity

**Fig 5:**
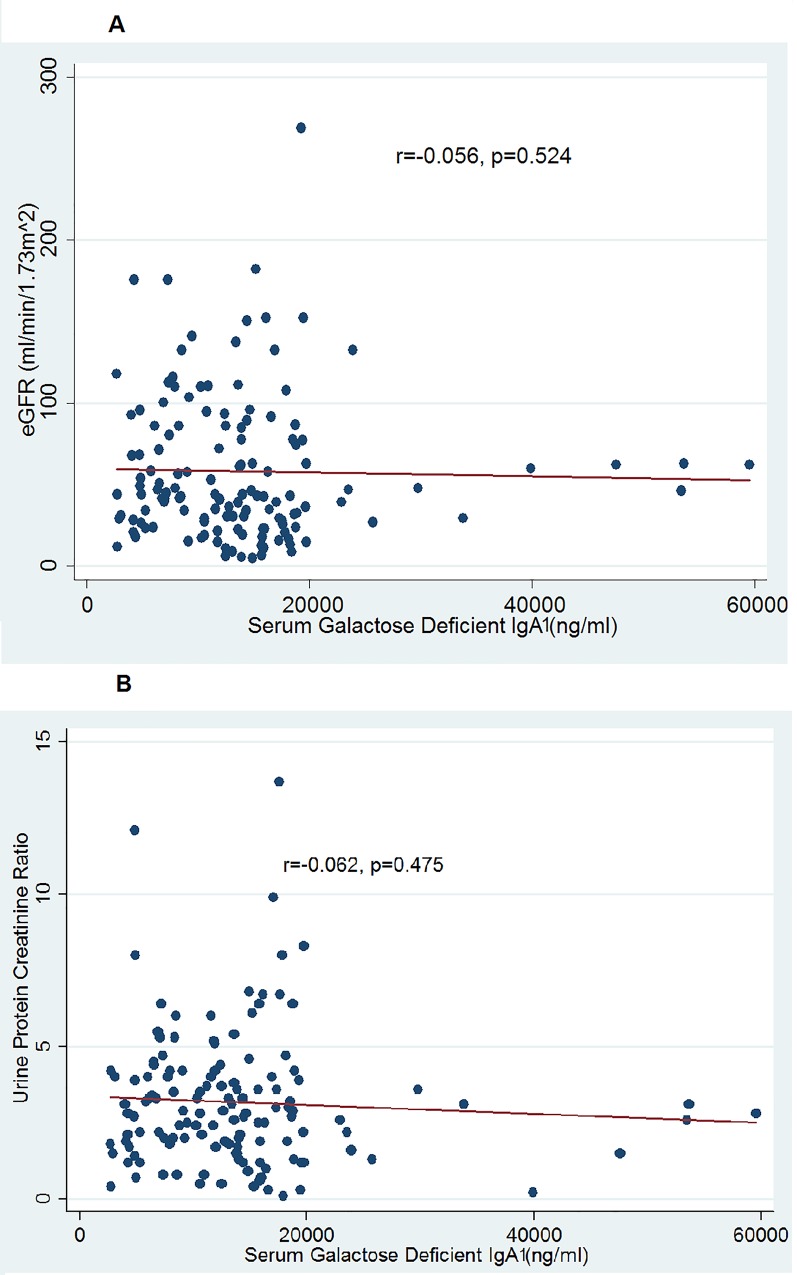
Co-relation of serum Gd-IgA1 with (5A) eGFR and (5B) urinary protein creatinine ratio.

**Fig 6:**
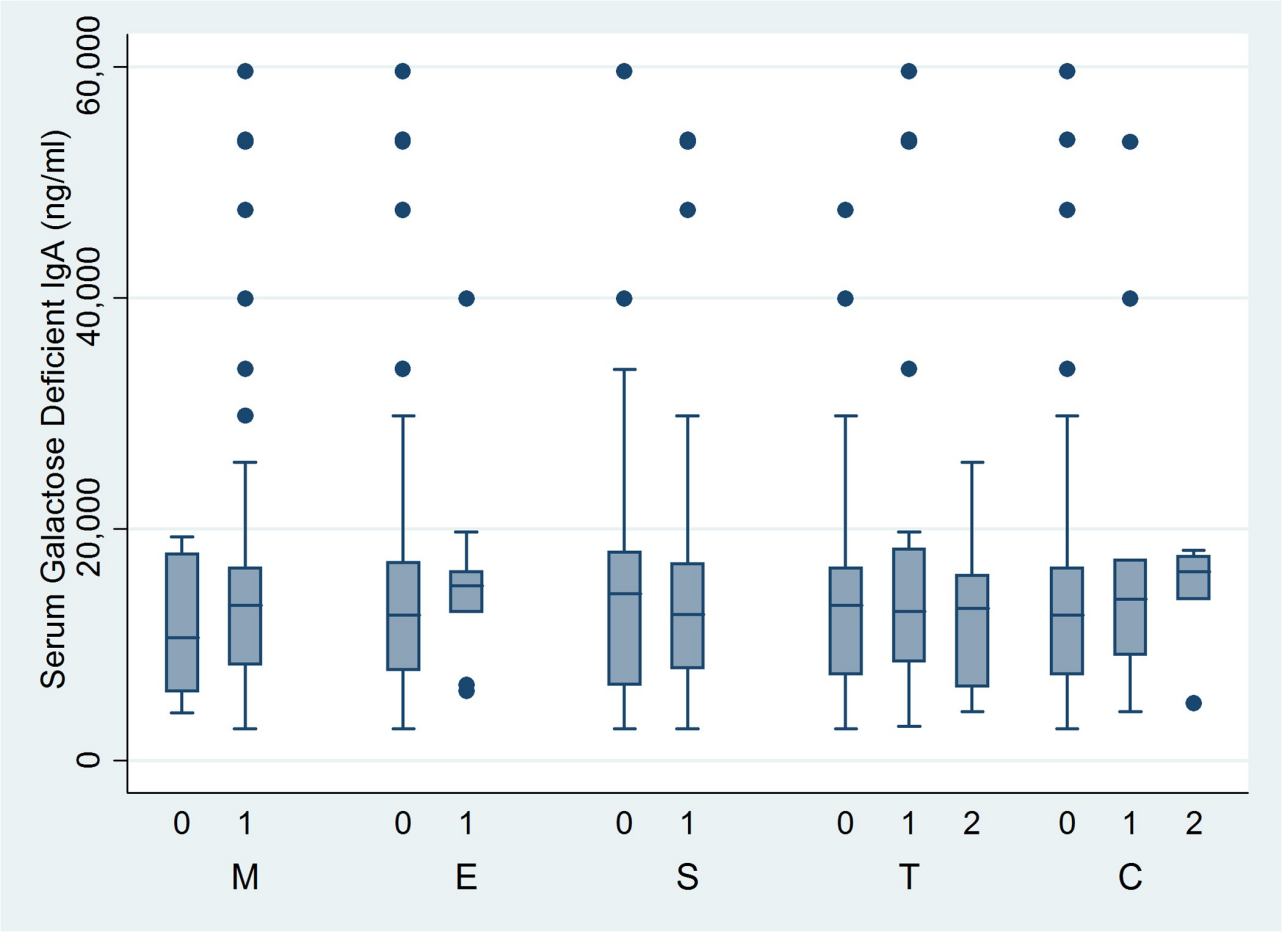
Serum Gd-IgA1 levels according to histologic severity of disease as per Oxford MEST-C criteria.

We then evaluated whether there was any association of this biomarker with surrogate markers of disease severity like eGFR and urinary PCR (Fig 5A and 5B) by Spearman correlation coefficient. The serum Gd-IgA1 level did not corelate with eGFR(r = -0.056,p = 0.524) and urinary PCR (r = -0.062, p = 0.475). The association of serum Gd-IgA1 with the histological disease severity was also studied based on the Oxford classification of IgA nephropathy (19). As shown in Table 3 and Fig 6, there were no association of the serum Gd-IgA1 levels of patients with their MEST-C scores on renal biopsy (M: Mesangial hypercellularity, E: Endocapillary proliferation, S: Segmental sclerosis, T: interstitial fibrosis and tubular atrophy, C: Crescents).

**Table 3:** Serum Gd-IgA1 levels according to the Oxford MEST-C score of IgA nephropathy.

MEST-CCharacteristics(n)	Serum Gd-IgA1 level(ng/ml)(Median, Range)	P
M0 (19)	10596.4 (4077.8,19328.0)	0.4038
M1 (117)	13410.2 (2723.2, 59603.8)	
E0 (126)	12561.9 (2723.2,59603.8)	0.2675
E1 (10)	15070.0 (5976.1,39924.0)	
S0 (43)	14408.0 (2743.5, 59603.8)	0.5554
S1 (93)	12619.7 (2723.2, 53679.3)	
T0 (79)	13410.2 (2723.2, 47603.6)	0.6930
T1 (38)	12891.4 (2944.4,59603.8)	
T2 (19)	13108.0 (4237.6, 25730.4)	
C0 (115)	12504.0 (2723.2 59603.8)	0.4964
C1 (15)	13941.3 (4237.6, 53452.8)	
C2 (06)	16262.8 (4852.6, 18160.0)	

47 patients reached the primary end point in this study of which 15 patients had progressed to ESRD and 2 had died (one had >30% decline in eGFR and one had progressed to ESRD). The probability of renal survival in the patients with higher(≥7982ng/ml) and lower serum Gd-IgA1 levels were 67.0%(95% CI:56.5, 75.5) and 80.1%(95% CI:62.7,90.0) at 12 months and 39.6%(95% CI: 17.8,60.8) and 62.9%(37.8, 80.2) at 48 months respectively. Though the disease progression was higher with poorer renal survival in patients with higher Gd-IgA1 values it was not statistically significant (HR-1.28,95% CI:0.63–2.59, p-0.486).

## Discussion

IgAN is a glomerular disease with a wide clinical spectrum ranging from mild incidentally detected disease to rapidly declining renal function. About half of these patients are known to have increased serum levels of Gd-IgA1 and Gd-IgA1 containing circulating immune complexes. Serum Gd-IgA1 has been extensively researched as a non-invasive biomarker for IgAN. Most of these studies have used the HAA-lectin based assay [12–15].

Now a new non-lectin based ELISA is available, which uses a unique monoclonal antibody to Gd-IgA1 molecule(KM55) and it has been validated against the lectin based assay and found to be a robust assay for detecting serum Gd-IgA1[18].So we used it to study the significance of serum Gd-IgA1 in Indian patients with IgAN. Though there have been multiple studies [12–17] evaluating the role of this biomarker in Caucasian, Japanese and Chinese populations, there is no published data in Indians patients. IgAN is believed to have a more severe disease phenotype in Indian patients with higher prevalence of proteinuric disease and rapid progression to end stage kidney failure [3–6].

Serum Gd-IgA1 levels were significantly higher in our patients of IgAN compared to controls who were healthy or had non-IgA glomerular diseases. At a cut-off value of serum Gd-IGA1≥7982.1ng/ml, the sensitivity of the assay for diagnosing IgA nephropathy compared to the healthy population was 74.3% and specificity was 72.0% with a positive predictive value of 87.8% and negative predictive value of 50.7%.We had a similar sensitivity but lower specificity compared to the study by Moldoveanu et al [12]. Multiple studies have shown similar results with serum Gd-IgA1 levels being higher in patients with IgAN compared to controls [13–16]. The distribution of the serum Gd-IgA1 values was skewed in our study suggesting that there is a considerable overlap between the patient and control populations which has also been observed by Placzek et al [20]. In this scenario this biomarker is unlikely to replace kidney biopsy for diagnosing IgAN. However in patients with milder disease or those who are reluctant to undergo an invasive biopsy procedure, it can be used as an initial screening assay. Those with high serum levels of Gd-IgA1 may then be counselled for kidney biopsy.

There was no correlation between the baseline serum Gd-IgA1 level and parameters of disease severity including eGFR and urine protein creatinine ratio. Moldoveanu et al [12] also did not find any significant correlation between serum Gd-IgA1(HAA-IgA) level and eGFR as well as urinary PCR. However, subsequent studies by Berthoux et al [21] and Zhao et al [22] have reported an association of high serum Gd-IgA1 levels with disease severity as well a disease progression. A study by Kim et al [23] found that serum levels of Gd-IgA1, total IgA1 and IgA-IgG in patients with IgAN vary with immunosuppression use. A systematic review of 22 studies across geographies [24] suggested that the serum Gd-IgA1 level may play a role in differentiating IgAN from other kidney diseases but it was not associated with the severity of the disease.

Very few studies have examined the association between serum Gd-IgA1 levels and histology of IgAN [25,26]. A recent study [25] done in 33 patients showed that the serum Gd-IgA1 concentration was significantly higher in patients with a higher Oxford classification score for segmental glomerulosclerosis (S1) or tubular atrophy/interstitial fibrosis(T1/2). But the serum levels did not correlate with clinical parameters such as proteinuria or eGFR in this study, which is similar to our findings. A previous study from China [26] before the Oxford classification era has also shown an association of serum Gd-IgA1 concentrations in patients with more severe histologic disease which was not observed in our patients.

Zhao et al found a correlation between the serum level of Gd-IgA1 and disease progression [22]. In our study, the renal survival at 12 and 48 months was lower in patients with higher serum Gd-IgA1 levels compared to those with lower levels, however this was not statistically significant. Further studies with a larger cohort and longer follow up are needed to define the prognostic significance of this biomarker.

We could not study the auto-antibodies to Gd-IgA1 which have been shown to have a better co-relation with disease severity and progression and the significance of Gd-IgA1 deposits in the kidney biopsies due to logistic constraints. Despite these limitations, our study is significant considering that the non-lectin based ELISA has been used for the first time in a reasonable number of patients to establish a cut-off for serum Gd-IgA1 levels in IgAN patients. Also it is the first study to address this issue in the Indian population in whom IgAN is known to have a poor outcome.

To, conclude, our study shows that serum Gd-IgA1 level has a high positive predictive value to differentiate patients with IgAN from other glomerular diseases but does not corelate with disease severity and progression. The simplicity and robustness of this newer non lectin based assay makes serum Gd-IgA1 an attractive candidate biomarker for IgAN especially in patients with mild disease.

## Supporting information

S1 File Clinical characteristics and serum Gd-IgA1 levels of patients and controls. (XLSX)
